# The challenge of learning a new language in adulthood: Evidence from a multi-methodological neuroscientific approach

**DOI:** 10.1371/journal.pone.0246421

**Published:** 2021-02-19

**Authors:** Sarah Steber, Sonja Rossi

**Affiliations:** 1 ICONE—Innsbruck Cognitive Neuroscience, Department for Hearing, Speech, and Voice Disorders, Medical University of Innsbruck, Innsbruck, Austria; 2 Department of Psychology, University of Innsbruck, Innsbruck, Austria; University of Florida, UNITED STATES

## Abstract

Being proficient in several foreign languages is an essential part of every-day life. In contrast to childhood, learning a new language can be highly challenging for adults. The present study aims at investigating neural mechanisms supporting very initial foreign language learning in adulthood. For this reason, subjects underwent an implicit semantic associative training in which they had to learn new pseudoword-picture pairings. Learning success was measured via a recognition experiment presenting learned versus new pseudoword-picture pairings. Neural correlates were assessed by an innovative multi-methodological approach simultaneously applying electroencephalography (EEG) and functional near-infrared spectroscopy (fNIRS). Results indicate memory-related processes based on familiarity and mechanisms of cognitive control to be present during initial vocabulary learning. Findings underline the fascinating plasticity of the adult brain during foreign language learning, even after a short semantic training of only 18 minutes as well as the importance of comparing evidence from different neuroscientific methods and behavioral data.

## 1. Introduction

Verbal communication displays one of the most essential cognitive functions in everyday social life. In order to communicate successfully, words and rules of a certain language have to be learned and implemented thoroughly. Infants and children master this developmental step with an astonishing effortlessness while as adults we tend to have more difficulties and are more challenged when learning a new language. As globalization grows however, being proficient in several languages gains more and more importance even at later stages of life. Language is a conglomerate of different abilities including phonology, prosody, semantics, syntax, and pragmatics. All of them contribute to a successful communication. However, one of the important steps during initial language acquisition is building up a vocabulary. Without knowing words and names for objects, we are not able to create sentences with meaning.

The *Revised Hierarchical Model* (RHM) suggests that in very initial foreign language learning, meaning of the new language (L2) is attained via already existing knowledge from the native language (L1) serving as a mediator and memory aid [1–3]. L1 information can be accessed lexically (via phonology of the word per se) as well as semantically (by the concept the individual subject has of a specific word) [1–3].

In the educational setting (e.g., school education), experimental designs on word learning therefore mostly rely either on a word-word (presenting the L2 word alongside with the respective translation in L1) or picture-word (presenting the L2 word alongside with a picture of an object representing the word from L1) learning paradigms. Behavioral research shows that both strategies seem to be beneficial depending on context, age of acquisition of L2, and learning strategy preference of the individual subject [4, 5].

In general, pictures seem to be easier to remember than words alone, as they provide both verbal (i.e., familiar objects can be named) as well as pictorial cues [5, 6] (e.g., *dual coding theory* [7, 8]). Following assumptions of the RHM, this picture superiority effect might fundamentally support foreign vocabulary learning, as pictures provide additional conceptual information beyond pure lexical and semantic cues of the L1 word that can be used as additional mnemonics. Indeed, such mnestic strategies seem to be commonly performed by individuals when learning a new language in an educational setting, as often mental images are created by the learners and then linked to the L2 word as a retrieval cue in order to create meaning [4, 9]. However, when building up a vocabulary in a natural environment (i.e., in a foreign country) without a concrete learning setting, such strategies are not necessarily applied in a conscious manner.

Nevertheless, we are able to learn a new language implicitly by using learning strategies such as *statistical learning*, which operates on the basis of probabilities of occurrence of specific rules. In this sense, everything that is more frequently combined and therefore common in the environment should be learnt. Usually, this occurs via mere passive listening without concrete instruction or feedback (for a recent review please refer to [10]). Literature suggests that statistical learning processes are strongly interwoven with memory encoding, storing, consolidating, and retrieval processes [11, 12]. When it comes to word learning, statistical learning mechanisms particularly come into play during *associative semantic learning*, that is, learning new words in combination with objects (e.g., [13]). Such a learning setting is crucial during childhood language acquisition [14, 15], but also during adulthood we are frequently confronted with a similar learning environment when learning a foreign language naturally. In contrast to children however, adults are already familiar with a variety of objects to which they have to assign new names. Therefore, adults are provided with a greater variability of conceptual and pictorial cues of the respective objects (based on personal experience) [16] as well as with lexical and semantic cues from the L1 (e.g., the L1 name of the object) [17, 18]. Overall, regardless of context (implicit natural setting or explicit educational setting), relying on objects seems to be very beneficial when it comes to language learning.

Most research regarding vocabulary learning in picture-word paradigms uses behavioral parameters. Neuroscientific evidence in regard is scarce. In order to investigate neuroscientific mechanisms of initial and implicit foreign vocabulary learning in adults, we therefore opted for the application of an associative semantic word-learning paradigm. The repeated presentation of the same congruous word-object pair intermixed with variable incongruous pairings allows the learner to familiarize “learned” (i.e., congruous) and to discard “new” (i.e., incongruous) associations. However, in contrast to most behavioral research, which uses real existing foreign language words or rules, we decided to use self-constructed pseudowords corresponding to foreign linguistic rules. This allows for uncovering initial foreign language learning processes that are not confounded by prior knowledge arising from the subjects’ native language or different linguistic proficiency levels acquired in a second language.

In the present study, neural processes were assessed via two neuroscientific methods that provide a quite ecologically valid learning setting. Functional near-infrared spectroscopy (fNIRS) was applied in order to reveal accurate topographical evidence about the involvement of brain areas. Temporally precise electrophysiological responses were assessed by means of electroencephalography (EEG). Both methods do not interfere with each other and are both soundless, thus being ideal for the investigation of acoustic linguistic stimuli. In general, neuroscientific studies showed that even when a foreign language is learned during adulthood, a high proficiency level can be attained while brain areas similar to those of the L1 are recruited [19–22].

It is known, that language is lateralized in the adult brain. The *Dynamic Dual Pathway Model* [23] provides a dichotomy of language functions and assigns these to different hemispheres. Segmental information such as phonology, lexico-semantics, and syntax predominantly recruits a fronto-temporo-parietal network of the left hemisphere while suprasegmental information such as prosody is mainly processed in homologous right hemispheric areas. This is in line with the *multi-time resolution hypothesis* [24] as well as the *dual-stream model* [25].

Phonological and lexico-semantic aspects play a central role during word learning. Single phonemes have to be identified and combined to phonological word forms that can afterwards be accessed in the mental lexicon. Only then, the meaning of a word can be retrieved. In general, phonological and lexico-semantic processing was found to elicit activations in a fronto-temporo-parietal network comprising predominantly the superior temporal gyrus (STG), middle temporal gyrus (MTG), angular gyrus (AG) as well as areas in the ventrolateral prefrontal cortex (vlPFC) including the inferior frontal gyrus (IFG) (for a review, see [26]). In language learning settings, both structural and functional changes were found by means of magnetic resonance imaging (MRI). In particular, grey matter increases of frontal and temporal areas could be attested with increasing L2 proficiency [27–31]. Frontal regions, including prefrontal areas as well as IFG, showed a specific importance in semantic retrieval and selection, which necessitates language control mechanisms [26, 32–37]. Temporal areas, in particular the MTG, were assigned an important role during long-term storage of conceptual-semantic knowledge [37, 38]. A larger reliance on language control areas, especially at the beginning of language learning (i.e., in low proficient learners), is postulated by the *Convergence Hypothesis* (CH) [39, 40]. During initial language learning, semantic retrieval in L2 is assumed to be more effortful and difficult, thus it necessitates increased controlled processes neurally reflected by enhanced frontal activations. This assumption was also confirmed by functional MRI studies revealing increased activations in frontal areas for low proficient learners that shifted to a more temporal recruitment with increasing L2 proficiency when semantic representations are better instantiated [41–43]. It should be noted, however, that the before mentioned studies trained participants over several days and months with multiple training sessions. Therefore, it is unclear whether similar activations arise for very initial vocabulary learning after only one short semantic training.

Electrophysiological studies investigating foreign language learning with an associative learning paradigm are scarce and mostly found modulations with respect to the N400 component. The N400 reflects lexico-semantic mechanisms and is sensitive to processing effort with the amplitude being larger when access to the mental lexicon is more difficult [44]. Dobel et al. used magnetoencephalography (MEG) to investigate a semantic associative learning paradigm [45]. Stimuli consisted of pseudowords as well as real words combined with pictures of real objects. First, the acoustic word/pseudoword was presented, followed by a real object after 200 ms. Adult subjects had to learn the correct associations from repetitive correct combinations intermixed with random combinations (i.e., distractors). During this training, subjects had to press a button whether the combinations matched or not but did not get any feedback. They were trained 20 min á day on 5 consecutive days. On each day, congruous and incongruous word-picture pairings were presented four times each. In order to assess neural changes elicited by the training, a semantic priming procedure was performed during which the acoustic word/pseudoword was presented at first, followed by the picture presentation after 700 ms. Subsequently, subjects had to indicate by button press whether the picture was a man- or nature-made object. Results showed a reduced activation after training for pseudowords approximating that of real words, which was interpreted as a reflection of successful access to the mental lexicon as well as an automatization process.

It should be noted that this study used pseudowords that conformed to linguistic rules of the subjects’ native language. Thus, subjects were already familiar with these rules pre-experimentally due to their lifelong experience with their native language. Similar reduced brain activation in frontal and temporal areas was also attested by a learning study [46] in which new pseudowords had to be learned together with the corresponding native language word. This reduction in activation was also interpreted as an approximation to a real word status. In a further MEG study with a similar training paradigm, Dobel, Lagemann, and Zwitserlood used pseudowords including a non-native phoneme completely unknown to the subjects [47]. Congruous and incongruous word-picture pairings were presented 8 times each per day. Interestingly this time, the magnetic N400 amplitude for pseudowords including a non-native phoneme increased after training. Therefore, these originally completely unknown pseudowords seemed to have lost the non-word status (usually indexed by a very small amplitude) and the integration in the lexicon started. A similar N400 modulation for native and non-native rules was also attested in a 3-day learning study [48] using a proto-semantic categorization task in which subjects had to learn to assign new pseudowords to an arbitrary category.

One important issue during language learning is the successful retrieval from memory. Several aspects were discussed in literature that elicit differential neural patterns and seem to play a crucial role during recognition tasks. In this regard, the differentiation of *familiarity* versus *recollection* has to be highlighted. The former describes a feeling of recency while the latter points to the retrieval of details of the learned items [49] (for a recent review in regard please refer to [50]). Furthermore, familiarity can be subdivided into *relative* and *absolute* familiarity [50]. Relative familiarity establishes when new information (e.g., never heard pseudowords) was presented recently in a learning context, for example by repetition. Absolute familiarity is built on pre-experimentally and already established information, thus life-long experiences (e.g., adults’ knowledge of the word *ball* is already present since childhood). Electrophysiologically, old/new effects (i.e., larger positive effects for learned/more familiar items) were attested during recognition memory tasks. However, relative and absolute familiarity processes were found to give rise to two distinct event-related potential (ERP) components: a frontally distributed FN400 for relative and a posteriorly distributed N400 for absolute familiarity (e.g., [51, 52]). Topographically, in particular the perirhinal cortex (PrC) was found to play a crucial role in this regard [53]. Familiarity judgements (e.g., deciding whether an item is familiar or not) were further found to be impacted by top-down regulation mechanisms such as allocation of attention and cognitive control [50]). Such top-down mechanisms relevant during the selection/decision of a correct response seem to be predominantly processed by the lateral prefrontal cortex with larger activations for correctly identified familiar items than incorrect decisions [49, 54–57].

In the present study, we constructed an associative learning paradigm combining statistical learning rules adopted in the studies by Dobel et al. [45, 47]. To assure a learning setting that reflects very initial word learning as naturally as possible, participants underwent an implicit semantic associative training without feedback at first. Immediately afterwards, a recognition experiment was conducted in order to evaluate explicit retrieval of previously learned word-picture pairings. However, we opted for several modifications compared to Dobel and colleagues [45, 47]: (1) we used only non-native pseudowords conforming to foreign phonotactic rules (i.e., permissible combinatory rules of phonemes in a specific language [58]), in order to maximize the degree of unfamiliarity of our acoustic material; (2) completely unknown/unfamiliar pseudowords and pictures of pre-experimentally familiar real objects were presented simultaneously during both training and recognition; (3) learning during semantic training was completely implicit (i.e., without any task); (4) in order to investigate very initial fast learning unaffected by a potential influence of overnight consolidation (e.g., [59]) we decided to train our subjects only on one day with the training lasting 18 minutes and congruous/incongruous pairings repeated six times each; (5) in order to evaluate neural changes elicited by the training as well as learning success, a recognition experiment was conducted in which learned and new combinations were presented and had to be explicitly identified by button press. These changes were intended to create an implicit associative learning training resembling very initial vocabulary acquisition in childhood (i.e., simultaneous combination of an object and its name without a task but including distracting combinations).

Apart from shedding light on the neural underpinnings of initial vocabulary learning in adulthood, the present investigation also allows for attesting the feasibility and impact of an implicit, short associative paradigm using foreign phonotactics on relative and absolute familiarity mechanisms. Based on previous findings, in the recognition experiment we expected to find either an FN400 or an N400 in the EEG, both with more positive amplitudes for congruous/learned pseudoword-picture pairings (PPPs) resembling the influence of either relative or absolute familiarity. For the fNIRS, we expect increased activations particularly in lateral prefrontal regions for congruous/learned compared to incongruous/new PPPs if top-down influences such as cognitive control are relevant during the decision/selection process.

The use of familiar pictures and completely unknown foreign pseudwords provides the opportunity to assess whether absolute familiarity (elicited by the presented pictures), relative familiarity (induced by unknown pseudowords) or even both may be the driving force during recognition of learned combinations. Furthermore, the innovative application of two neuroscientific methods bears the potential to provide detailed insights into the underlying neural processes.

## 2. Material and methods

The present study was approved by the Ethics Committee of the Medical University of Innsbruck, Austria (no. AN2016-0204 366/4.17). Participants gave written informed consent.

### 2.1 Participants

In total, 34 healthy adults (13 male) participated in the present study. Only those subjects were included in the final statistical analyses that performed well during the recognition experiment (at least 60% of all items correctly identified, i.e., 36 of 60 items). Due to this inclusion criterion, one subject had to be excluded from the study sample. The remaining 33 subjects entered EEG analyses. Data of two further subjects had to be excluded from fNIRS analyses due to technical problems during measurements. All 33 participants were 34 years old on average (range: 22–50 years). All were healthy, had no neurological disorders, did not suffer from hearing or visual impairments and no prematurely born subjects were included in the study. Furthermore, participants reported no severe deficits in language development during childhood as well as no deficits in reading and speaking in general (e.g., dyslexia).

Participants were raised monolingually with German as their native language and lived in Austria at time of testing. Some studies showed that being raised bilingually from birth alters brain development and especially impacts cognitive abilities relevant for language learning [60–63]. In the present study, all participants learned their first foreign language (i.e., English n = 29, Italian = 2, French = 1, Latin n = 1) at a mean age of 9 years (range: 7–12 years). 30 subjects learned a second foreign language (i.e., French n = 14, Italian = 7, Spanish n = 5, English n = 4) at a mean age of 15 years. 19 subjects learned a third foreign language (i.e., Italian n = 6, Latin n = 5, French n = 4, Spanish n = 2, Greek n = 1, Norwegian n = 1) at a mean age of 18 years. 11 subjects came in contact with a fourth foreign language (Spanish n = 5, Italian n = 4, Japanese n = 1, Latin n = 1) at a mean age of 22 years. Importantly, subjects had no knowledge in Slavic languages. This was strictly controlled for as the stimulus material included linguistic cues of the Slovak language, which should be unfamiliar to the participants. Self-reported proficiency levels of each learned language were obtained from each participant on 6-point Likert scales for proficiency in hearing, reading, writing, and speaking (1 = like mother tongue, 6 = mostly forgotten). Please note that most participants reported to have no remaining knowledge in the third and fourth foreign languages. Subjects of the present study showed to have quite high mean language proficiency levels: German (n = 33): hearing = 1.0, reading = 1.06, writing = 1.18, speaking = 1.15; first foreign language (n = 33): hearing = 2.18, reading = 2.15, writing = 3.15, speaking = 2.82, second foreign language (n = 30): hearing = 3.8, reading = 3.67, writing = 4.63, speaking = 4.43, third foreign language (n = 19): hearing = 5.47, reading = 5.21, writing = 5.74, speaking = 5.58; fourth foreign language (n = 11): hearing = 5.36, reading = 5.27, writing = 5.64, speaking = 5.55.

We assessed handedness in all subjects using the Oldfield Handedness Inventory [64]. All participants were right-handed with a mean score of 87.55 (range: 66.7–100). Level of education was rather high in the present study (university degree: n = 22; high school: n = 5; compulsory schooling: n = 6).

Additionally, we conducted the d2 Test: Concentration Endurance Test [65] which is a time test in order to measure attention and concentration capabilities. All participants in the present study were in the average (n = 6) or above average (n = 27) range (mean d2 score = 493.3; min = 333; max = 642; PR range = 50–99.9).

### 2.2 Material

Since the aim of the present study was to investigate initial foreign language learning in adulthood, we structured stimuli in such a way that they would be as unfamiliar as possible to the participants. In total, 30 pseudowords were constructed. All pseudowords consisted of CCVCV (consonant-consonant-vowel-consonant-vowel) combinations. Onset consonant clusters conformed to phonotactic rules of the Slovak language, which belongs to the Slavic languages. The Slovak language has proven to be very suitable for foreign language study designs with German native speakers since it provides a greater variability of consonant combinations in word onsets than the German language [48, 66, 67]. Thus, the linguistic rules of pseudowords were unfamiliar to all participants.

For all 30 pseudowords, the following onset consonant clusters typical for Slavic languages and non-existent in German were chosen: /bd/, /dw/, /tm/, /fp/, /fn/. Six bisyllabic pseudowords were formed per onset cluster (e.g., fpogo, bdafa, tmipi), while additionally ensuring that the frequency of vowels and consonants in all pseudowords was equally distributed.

The voice recordings took place in an anechoic chamber (Laboratory for Psychoacoustics at the Department of Hearing, Speech, and Voice Disorders of the Medical University of Innsbruck) and were performed by a female speech scientist. Pseudowords were spoken in a neutral prosody. Additionally, all pseudowords were spoken with a trochee stress pattern on the first syllable corresponding to the most frequent stress pattern in German and thus, not further introducing another foreign language factor to the material. All stimuli were recorded at 44 kHz and 16 bit sampling rate. Afterwards, the acoustic stimuli were edited using the editing programme *Audacity* (www.audacityteam.org). This procedure predominantly included cutting, inserting a short silence period of 30 ms at the onset and offset of each pseudoword, and normalizing pitch and loudness.

Furthermore, 30 pictures of common objects were needed for the experiments. Therefore, we narrowed down the standardized picture pool by Rossion and Pourtois [68] to 47 colored pictures of real objects whose descriptions consisted of bisyllabic words to match the selected pseudowords (e.g., Bir-ne—engl. pear). Furthermore, all picture names did not contain any consonant clusters at word onsets in order to not confound with phonotactic rules of pseudowords used in the present study. The pictures were evaluated by 30 independent but age- and gender-matched raters via an online survey (www.soscisurvey.de). Participants had to name all objects and those 30 pictures were chosen that had the highest naming correspondence. The final 30 pictures were assigned to 5 categories: animals (n = 8), music instruments (n = 2), cutlery (n = 3), food items (n = 5), and other (n = 12).

Finally, pseudoword-picture pairings (PPPs) for the semantic association training were constructed. For this, each pseudoword was assigned to one colored real object in order to create 30 congruous pairings (i.e., PPPs that should be learned during the training; e.g., /fpogo/ with Birne—engl. pear). All congruous PPPs met the following criteria: (1) pseudoword and object name did not start with the same letter (e.g., /fpogo/ and Birne) and (2) pronunciations of both pseudoword and object name did not sound similar. Then, six additional but different pictures were assigned to each pseudoword to generate six incongruous pairings per pseudoword (i.e., PPPs that served as distractors during the semantic training, e.g., /fpogo/ with Leiter–engl. ladder, Hase–engl. rabbit, Sofa–engl. sofa, Kette–engl. necklace, Löwe–engl. lion, and Messer–engl. knife). Then, an additional seventh incongruous PPP was constructed per pseudoword for the recognition experiment in order to create a picture-pseudoword combination not presented in the semantic training (e.g., /fpogo/ and Eule–engl. owl) and therefore serving as a new/unlearned pairing in contrast to the congruous/learned pairings.

**Fig 1:**
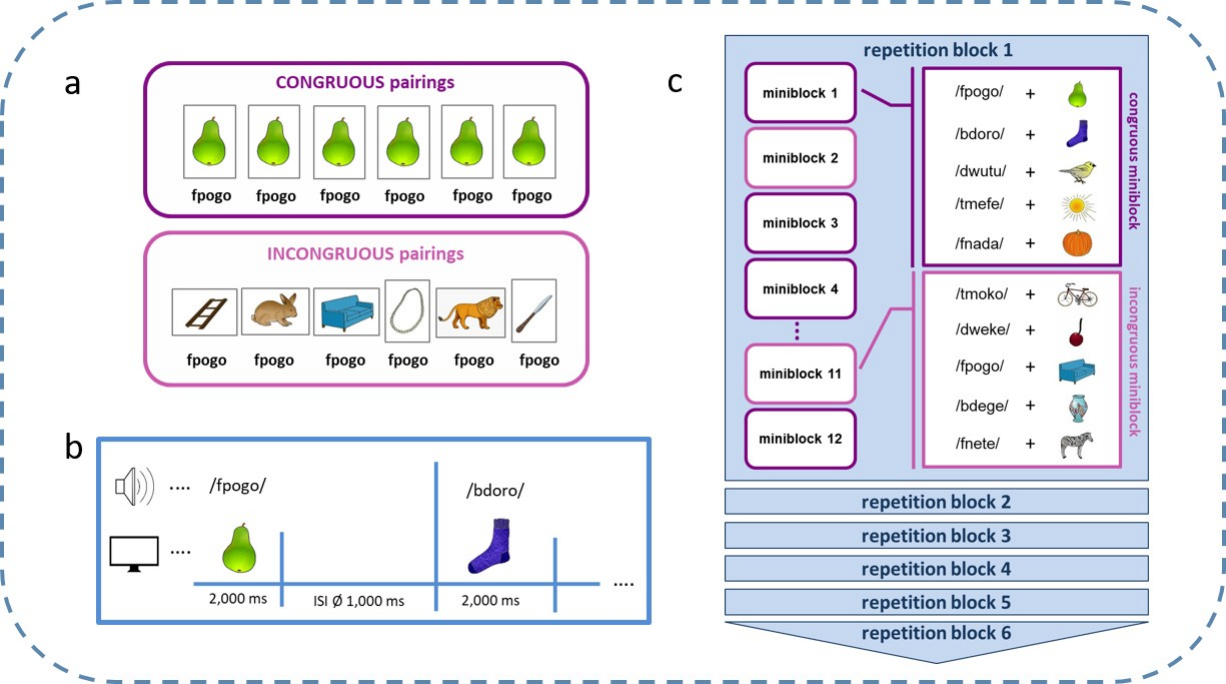
Design of implicit semantic training **a.** Example for one congruous pseudoword-picture pairing (PPP) (/fpogo/ with pear) pseudorandomly repeated six times during training and its additional six incongruous pairings (distractors). **b.** Example for presentation sequence of PPPs **c.** Illustration of the block design used for the semantic training in the present study. Images were taken from [68] with image courtesy of the authors as well as from license-free databases.

The following criteria were met during the construction of all seven incongruous PPPs: (1) pseudoword and object name did not start with the same letter (e.g., /fpogo/ and Leiter–engl. ladder), (2) object names of incongruous pairings did not start with the same letter as the object name of the respective congruous pairing (e.g., Birne and Leiter, Hase, Sofa etc.), (3) no more than 2 out of 7 object names started with the same letter, (4) pseudoword and object name did not sound similar, (5) object names of all 7 incongruous pairings were not allowed to sound similar (e.g., Hase–engl. rabbit and Hose–engl. trousers) or look similar (e.g., zebra and donkey). The following additional rules applied for picture categories: (1) in all 7 incongruous pairings, no more than 1 music instrument, 1 cutlery, 2 food items, and 3 animals were allowed, (2) if 3 objects belonged to the animal category, the third animal object had to be assigned to the seventh PPP (i.e., the new pairing for the recognition experiment), (3) if the object of the congruous pairing belonged to categories music instrument, cutlery, or food items, no other picture out of these categories was allowed for the respective incongruous pairings, and (4) if the object of the congruous pairing belonged to the animal category, only one additional object of this category was allowed in all 7 respective incongruous pairings. All in all, 30 congruous PPPs were constructed with seven incongruous PPPs respectively, resulting in a total number of 240 pseudoword-picture combinations. Please refer to Fig 1 for an example of PPP construction.

### 2.3 Procedure

Neural activity was assessed simultaneously by means of electroencephalography (EEG) and functional near-infrared spectroscopy (fNIRS). The former method excellently tracks fast processing mechanisms in the millisecond range, whereas the latter provides a good spatial resolution indicating the underlying brain areas recruited. This multi-methodological approach has proven to be highly beneficial for investigating acoustic stimuli, as both methods are soundless, do not interfere with each other, and provide a quite natural setting [69].

During the experiments, subjects sat on a chair 1 m in front of a computer monitor. To enable a simultaneous measurement of both methods, subjects wore elastic EEG caps (EasyCap GmbH, Herrsching, Germany) in which both EEG electrodes as well as fNIRS optodes were integrated. Pseudowords were acoustically presented via loudspeakers at an intensity of 70 dB. The pictures of colored objects were shown on the computer screen simultaneously to the auditory presentation of pseudowords.

#### 2.3.1 Implicit semantic training

At first, an implicit semantic training in form of a picture-word-association training without feedback was conducted in all subjects. Subjects did not have to perform any specific task in order to capture implicit learning mechanisms. The presentation of each PPP lasted 2 s interspersed with a constant inter-stimulus-interval (ISI) of 1 s. Over the course of this training, all 30 pseudowords were presented six times with the same picture (congruous pairings) as well as six times with six different pictures (incongruous pairings, distractors). All 240 PPPs were presented in a pseudorandomized fashion using a block design meeting the following criteria: 6 large *repetition blocks* in total, each containing the congruous pairing of each pseudoword (e.g., /fpogo/ with the pear) as well as one of the six incongruous pairings (e.g., /fpogo/ with the sofa). Each repetition block consisted of 12 *miniblocks*. Then again, each miniblock contained five consecutive picture-word combinations belonging either to only the congruous or the incongruous pairing condition. Miniblocks within one large repetition block were pseudo-randomized by ensuring that no more than three congruous or incongruous miniblocks were presented in succession. Please refer to Fig 1 for a better understanding of the procedure. In total, 4 pseudorandomization versions were used for the semantic training which lasted 18 minutes.

#### 2.3.2 Recognition experiment

In the recognition experiment, which followed immediately after the semantic training on the same testing day, actual retrieval from memory by means of a picture-word-identification task was tested. All 30 *learned* PPPs (i.e., congruous PPPs from the semantic training) were presented again. In addition, each pseudoword was presented again in a new picture-pseudoword combination that did not appear in the semantic training. (i.e., *new* PPP). Please note that pictures in new PPPs were taken from the same object picture pool as in the semantic training, meaning that both pseudowords and pictures were now familiar to the subjects, but the respective combination of a specific pseudoword with a specific picture was new. Thus, in the recognition experiment, each pseudoword was presented twice, one time in a previously learned pairing and one time in a new pairing. All PPPs were presented in a pseudorandomized manner with the following criteria: (1) maximally three consecutive learned or new PPPs in succession, (2) at least three different pictures between the same object, and (3) at least three different pseudowords between words with the same onset clusters.

**Fig 2:**
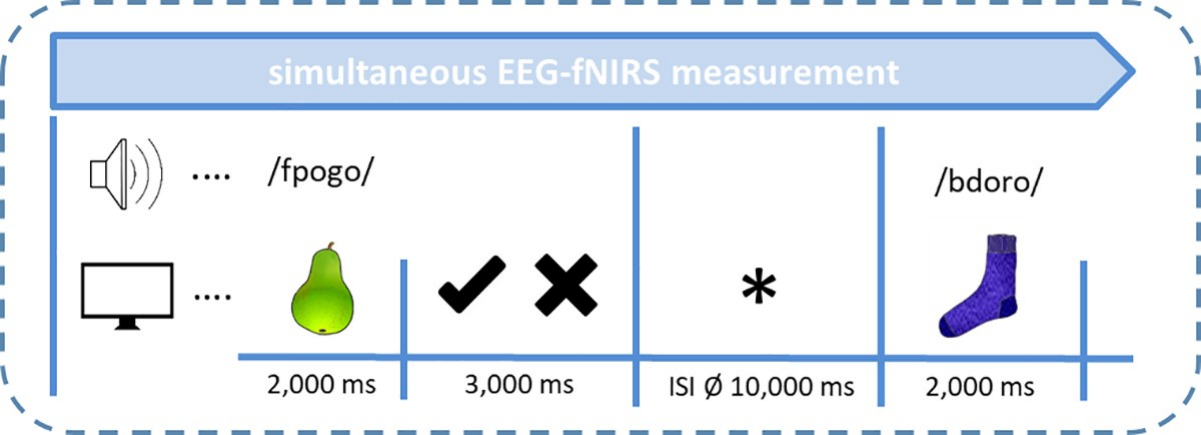
Experimental design of recognition experiment. Images were taken from [68] with image courtesy of the authors as well as from license-free databases.

PPPs were presented for 2 s. Afterwards subjects had to explicitly press a left or right button (counterbalanced across subjects) whether they recognized the previously learned picture and pseudoword combination or not. A visual signal prompted the button press for maximally 3 s. After each trial, a variable inter-stimulus-interval (ISI) (mean duration: 10 s, range: 6–14 s) was integrated (cf. Fig 2). By introducing this variable ISI, the experimental design was adjusted to the requirements of the rather slow hemodynamic response measured by the fNIRS. Usually, vascular responses reach their maximum at around 5 s after stimulus presentation with the activation returning to baseline after 15–20 s [70]. Therefore, variable ISIs prevent hemodynamic responses from overlapping systematically. In total, 4 pseudorandomization versions were used for the recognition experiment which lasted 14 minutes.

#### 2.3.3 Follow-up behavioral production test

In order to test whether subjects are able to successfully produce previously learned pseudowords and assign them to their respective object picture, we conducted a follow-up behavioral experiment. Participants (n = 29, 11 male, mean age: 33.9 years) matched with respect to all inclusion criteria, but not participating in the neuroscientific experiments, were confronted with both the semantic training as well as the recognition experiment. Right after the recognition experiment, they were then provided with all 30 object pictures and were asked to produce the respective corresponding pseudoword to each picture (i.e., learned PPP). In line with the neuroscientific measurements, only those subjects were included in final analyses that were able to at least identify 60% of all PPPs correctly in the recognition experiment. This resulted in 24 analyzed subjects. Furthermore, subjects were asked about the mnemonic strategies adopted during solving this task.

### 2.4 EEG recordings

**Fig 3:**
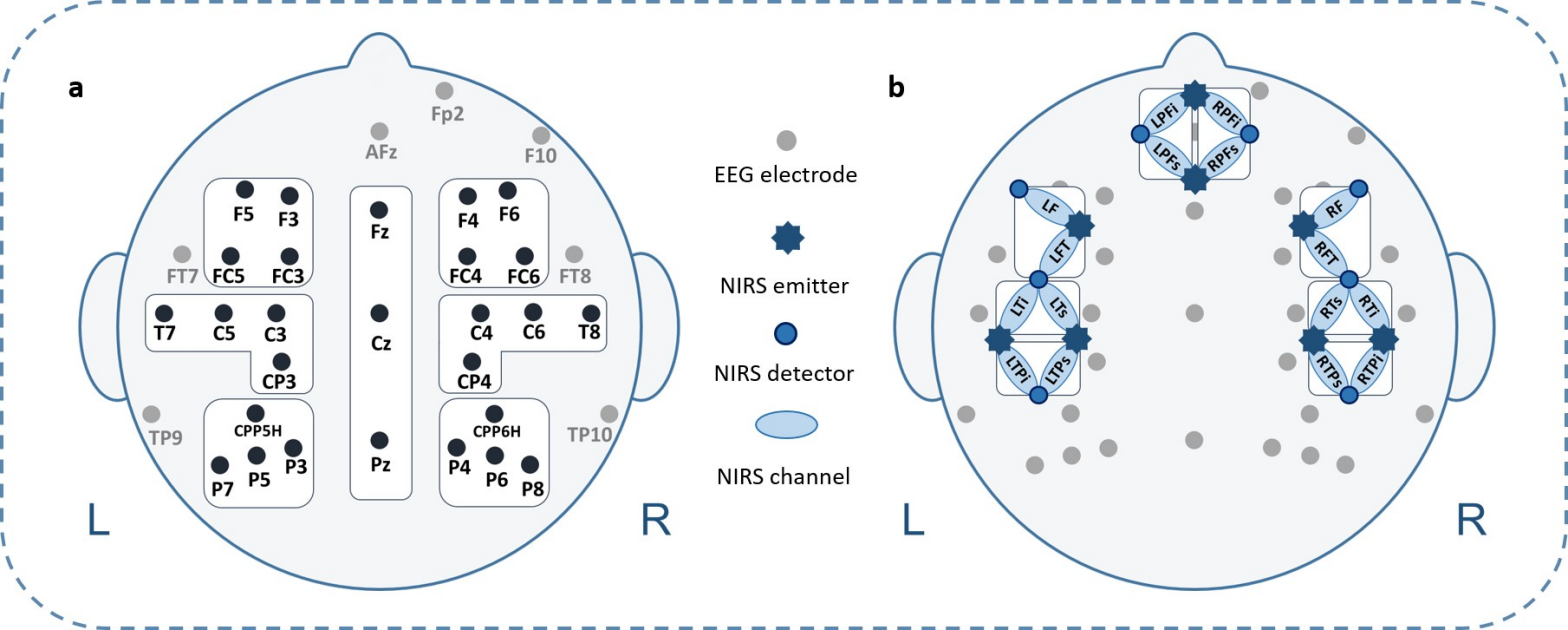
Simultaneous EEG electrodes and fNIRS channel placement. **a.** EEG electrode configuration including regions of interest (ROIs). **b.** fNIRS channel arrangement: stars indicate 8 fNIRS light emitters; dots indicate 8 fNIRS detectors; ellipses indicate fNIRS channels; channels cover prefrontal inferior (PFi), prefrontal superior (PFs), frontal (F), fronto-temporal (FT), temporal inferior (Ti), temporal superior (Ts), temporo-parietal inferior (TPi), and temporo-parietal superior (TPs) brain regions, for both hemispheres respectively. Additionally, all 8 left-hemispheric fNIRS channels are marked with an L; all 8 right-hemispheric fNIRS channels are marked with an R. Each two adjacent channels were combined to generate 4 ROIs per hemisphere.

EEG was recorded from 32 AgAgCl active electrodes (BrainProducts GmbH, Gilching, Germany) placed into an elastic EEG cap at the following positions: F5, F3, FT7, FC5, FC3, T7, C5, C3, CP3, CPP5H, P7, P5, P3, F4, F6, FC4, FC6, FT8, C4, C6, T8, CP4, CPP6H, P4, P6, P8, Fz, Pz, and Cz (cf. Fig 3). Vertical and horizontal electrooculogram were recorded above and next to the right eye with electrodes FP2 and F10. An electrode (TP9) at the left mastoid served as online reference, while an electrode at the right mastoid (TP10) was recorded for further re-referencing during offline analyses. Position AFz served as ground electrode. Electrode impedance was kept below 10 kΩ (actiCAP Control, Brain Products GmbH, Gilching, Germany). The EEG signal was measured by means of BrainVision Recorder (Brain Products GmbH, Gilching, Germany) software with a sampling frequency of 1000 Hz (amplified between 0.016–450 Hz) and filtered before digitalization by means of the analog/digital converter with an upper cut-off of 450 Hz (24 db/oct) to prevent aliasing.

### 2.5 fNIRS recordings

Vascular changes were measured by means of functional near-infrared spectroscopy. With this method, concentration changes of both oxygenated [oxy-Hb] and deoxygenated hemoglobin [deoxy-Hb] in cortical brain areas can be assessed by emitting light in the near-infrared spectrum to the biological tissue. Calculations of concentration changes in both hemoglobins are based on a modified Beer-Lambert law [71]. The physiological basis of fNIRS is the neurovascular coupling: an increased activation in a brain region leads to several vascular and metabolic changes. It is known that vasodilation leads to a local increase in blood volume demanding more oxygen and glucose, which in turn leads to an increase in regional cerebral blood flow and an increase in regional blood flow velocity [72, 73]. In consequence, the color of the blood changes. The blood flow increase overcompensates oxygen consumption and elicits a focal hyperoxygenation resulting in an increase in oxygenated hemoglobin [oxy-Hb] as well as a decrease in deoxygenated hemoglobin [deoxy-Hb] [74]. [Deoxy-Hb] is inversely correlated to the BOLD signal measured by functional magnetic resonance imaging (fMRI) (for more details see [75, 76]).

The used fNIRS system (NIRScout, NIRx Medizintechnik GmbH, Berlin, Germany) sends wavelengths at 760 and 850 nm in a cw-mode and recorded data at a sampling rate of 7.81 Hz. In total, eight light emitters and eight light detectors were used to assess activations over bilateral fronto-temporo-parietal brain areas. Interoptode distance was 3.5 cm. This emitter-detector configuration allowed the assessment of 8 channels per hemisphere, covering prefrontal inferior (PFi), prefrontal superior (PFs), frontal (F), fronto-temporal (FT), temporal inferior (Ti), temporal superior (Ts), temporo-parietal inferior (TPi), and temporo-parietal superior (TPs) brain regions (cf. Fig 3).

### 2.6 Data analyses

#### 2.6.1 Behavioral task performance during recognition experiment

Behavioral parameters of performance such as percentage of correctness of PPP identification and reaction times for both conditions (new and learned PPPs) were analyzed. In order to investigate differences between learned versus new PPPs, paired *t*-tests were applied for both performance and reaction times.

#### 2.6.2 EEG data

EEG data was filtered offline with a 30 Hz low pass Butterworth zero phase filter (high cutoff: 30 Hz; slope: 12 dB/oct). Data was then segmented from -200 ms to 1500 ms with 0 ms representing the time point of the PPP onset. An ocular correction based on the Gratton & Coles algorithm [77] was applied to correct vertical eye movement artefacts. Overly contaminated channels were rejected manually from each segment by inspecting each segment visually for artefacts. Only subjects in whom at least 50% of all segments per condition (learned versus new pairings) in at least 15 of all 29 electrodes survived this procedure were included in the final analyses. This criterion applied to all 33 subjects, thus no participant had to be excluded from statistical analyses. In the next steps, data was re-referenced to averaged mastoids (TP9, TP10) and a pre-stimulus baseline of 200 ms was applied. For the EEG, only correctly identified PPPs (i.e., learned PPP was correctly marked as “known”; new PPP was correctly marked as “unknown”) entered final statistical analyses.

Event-related brain potentials (ERPs) were extracted by averaging the segments for each subject and each condition (learned PPP vs. new PPP). In addition, a 50-ms-analysis was performed in order to select the time windows for final statistical analyses. This analysis included paired-sampled *t*-tests on each electrode between learned and new PPPs in consecutive 50 ms steps between 100 and 1500 ms. Results from this analysis as well as visual inspection of the grand averages revealed 650–800 ms and 800–1300 ms to be the time windows indicating differences between conditions and therefore were selected for further statistical analyses.

Since the topographical localization of EEG is only rough, we decided to perform the final statistical analyses on 6 regions of interest (ROIs). The following lateral ROIs were defined for statistical EEG analyses: left frontal (F3, F5, FC3, FC5), right frontal (F4, F6, FC4, FC6), left temporo-parietal (C3, C5, T7, CP3), right temporo-parietal (C4, C6, T8, CP4), left parietal (CPP5H, P3, P5, P7), and right parietal (CPP6H, P4, P6, P8) (cf. Fig 3). Midline electrodes (Fz, Cz, Pz) were analyzed separately. We then performed a three-way ANOVA with the within-subject factors Condition (learned vs. new PPP), Hemisphere (left hemisphere vs. right hemisphere), and Region (frontal vs. temporo-parietal vs. parietal) for lateral ROIs and a two-way ANOVA with Condition and Region (Fz vs. Cz vs. Pz) for midline electrodes. In order to check whether the correct identification of pseudoword-picture pairings has an impact on neurophysiological processes, we performed these ANOVAs for both correctly identified learned and new PPPs as well as for all presented PPPs (correctly and incorrectly identified), irrespectively of identification performance. Whenever a main effect of Condition or an interaction with Condition reached significance, post-hoc *t*-tests were subsequently performed. Significance level was set at *p*≤.050 and adjusted with the False Discovery Rate procedure [78]. Corrected significance according to Greenhouse and Geisser [79] was applied whenever the degrees of freedom exceeded 1.

#### 2.6.3 fNIRS data

In order to analyze concentration changes (mmol/l) of [oxy-Hb] and [deoxy-Hb], the collected reflected light was transformed by means of the modified Beer-Lambert function [71]. Exclusion of artefacts of each participant was performed manually. Artefacts (e.g., abrupt changes) were removed by a linear interpolation approach. A 0.4 Hz low pass filter (Butterworth, third order) was applied to attenuate high-frequency artefacts mainly arising from heartbeat. Next, data were correlated with a predictor generated by convolving the boxcar function of the stimulus design including both conditions (new vs. learned PPPs) with the canonical hemodynamic function [70, 80] peaking at 5 s. Data were then fed into a general linear model approach to obtain beta-values for each condition as well as for each of the two hemoglobins. Statistical analyses were performed on the beta-values of both [oxy-Hb] and [deoxy-Hb]. Please note that both a decrease in [deoxy-Hb] as well as an increase in [oxy-Hb] are considered as reflections of increased activation, thus we report both hemoglobins separately [75, 81].

Every two adjacent fNIRS channels were combined resulting in the following 4 regions of interest (ROIs) (left (L) and right (R) hemisphere respectively): prefrontal (LPFi, RPFi, LPFs, and RPFs), frontal (LF, RF, LFT, and RFT), temporal (LTi, RTi, LTs, and RTs), and temporo-parietal (LTPi, RTPi, LTPs and RTPs) (cf. Fig 3). We performed a three-way ANOVA with the within-subject factors Condition (learned vs. new PPP), Hemisphere (left hemisphere vs. right hemisphere), and Region (prefrontal vs. frontal vs. temporal vs. temporo-parietal) for [oxy-Hb] and [deoxy-Hb], separately. In order to check whether the correct identification of pseudoword-picture pairings has an impact on vascular processes, we performed these ANOVAs for both correctly identified learned and new PPPs as well as for all presented PPPs (correctly and incorrectly identified), irrespectively of identification performance. As this three-way ANOVA unfortunately did not yield any significant main effect or interaction, we subsequently performed a two-way ANOVA with the within-subject factors Condition (learned vs. new PPPs) and Hemisphere (left hemisphere vs. right hemisphere) on 4 regions (prefrontal, frontal, temporal, and temporo-parietal) for [oxy-Hb] and [deoxy-Hb], separately. Again, this analysis was performed for correctly identified PPPs as well as for all presented PPPs. Whenever a main effect of Condition or the interaction between Condition and Hemisphere reached significance, post-hoc *t*-tests were subsequently performed. Significance level was set at *p*≤.050 and adjusted with the False Discovery Rate procedure [78]. Corrected significance according to Greenhouse and Geisser [79] was applied whenever the degrees of freedom exceeded 1.

#### 2.6.4 Follow-up behavioral production test

Production rate was computed as follows: each pseudoword was split up in its 5 phonemes (e.g., b–z–o–p–o). For each correctly produced phoneme, 1 point was assigned. A total of 150 points could be achieved in this production test (30 PPPs, each pseudoword consists of 5 phonemes, 30x5 = 150). Subsequently, this rate was converted into percentages.

## 3. Results

### 3.1 Task performance and reaction times

All participants identified at least 60% of all 60 PPPs (30 learned and 30 new PPPs) correctly as known or unknown (mean 76.7%, range: 63.3–93.3%). Overall, a mean number of 20.3 out of 30 (range 15–29) learned PPPs (67.8%) was estimated correctly as known, while a mean number of 25.7 (range 20–30) new PPPs (85.6%) was rated correctly as unknown. A paired *t*-test between percentage of correctly identified learned versus new PPPs revealed a highly significant difference [*t*(32) = -6,453, *p* < .0001] indicating a better selection performance for new pairings.

Evaluation of reaction times (rt) revealed no significant differences between correctly identified learned and new PPPs (mean rt for learned: 1015.2 ms; mean rt for new: 965.6 ms).

### 3.2 EEG results

#### 3.2.1 EEG results for all pseudoword-picture pairings

The ANOVA did not result in any significant main or interaction effect for both time windows.

#### 3.2.2 EEG results for correctly identified pseudoword-picture pairings

**Fig 4:**
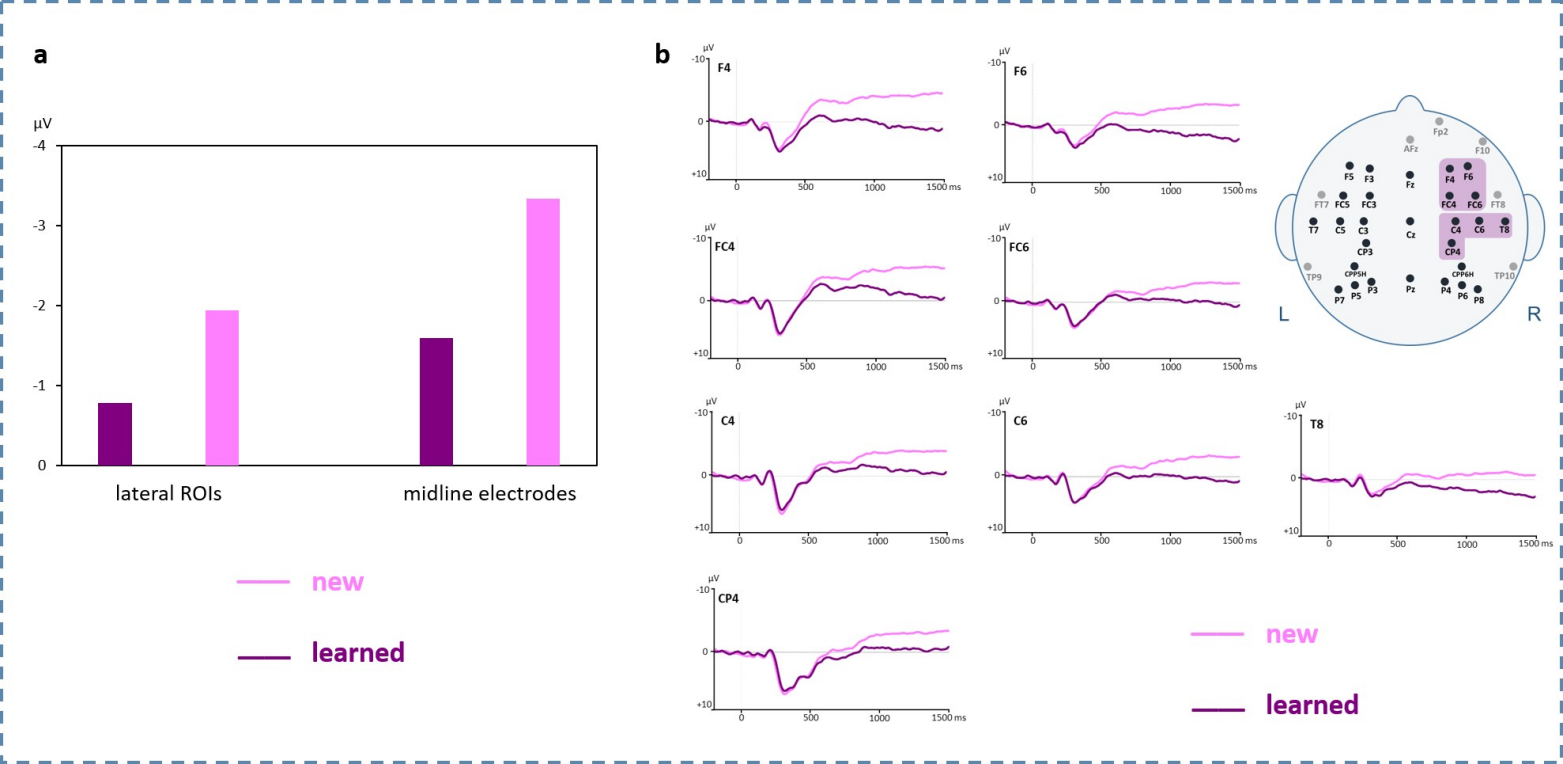
ERP results for new compared to learned pseudoword-picture pairings. **a.** Bar charts for time window 650–800 ms merged for all lateral ROIs (left) and for all midline electrodes (right). **b.** Grand averages for right frontal and right temporo-parietal electrodes. Significant differences were found on these electrodes between 800–1300 ms. Negative polarity is plotted upwards. An 8 Hz low-pass filter was applied for presentation purposes only.

For time window 650–800 ms, a three-way ANOVA (Condition x Hemisphere x Region) revealed a significant main effect for Condition on lateral ROIs [*F*(1,32) = 5.450, *p* = .026; η_p_^2^ = .146] showing larger negative amplitudes for new than learned PPPs (6 lateral ROIs merged) (see Fig 4). Furthermore, a significant main effect for Condition was found on midline electrodes (Fz, Cz, Pz) pointing in the same direction [*F*(1,32) = 6.492, *p* = .016; η_p_^2^ = .169].

For time window 800–1300 ms, the same three-way ANOVA (Condition x Hemisphere X Region) showed interaction effects for Condition x Hemisphere [*F*(1,32) = 6.772, *p* = .014; η_p_^2^ = .125] and for Condition x Hemisphere x Region [*F*(2,64) = 5.149, *p* = .012, η_p_^2^ = .139] on lateral ROIs. Post-hoc *t*-tests revealed significant differences on right frontal (F4, F6, FC4, FC6) [*t*(32) = -3.164, *p* = .003; FDR corrected at *p*≤.008] and temporo-parietal (C4, C6, T8, CP4) [*t*(32) = 2.530, *p* = .017; FDR corrected at *p*≤.017] ROIs with larger negativities for new compared to learned PPPs. No significant effects were found on midline electrodes.

### 3.3 fNIRS results

#### 3.3.1 fNIRS results for all pseudoword-picture pairings

**Fig 5:**
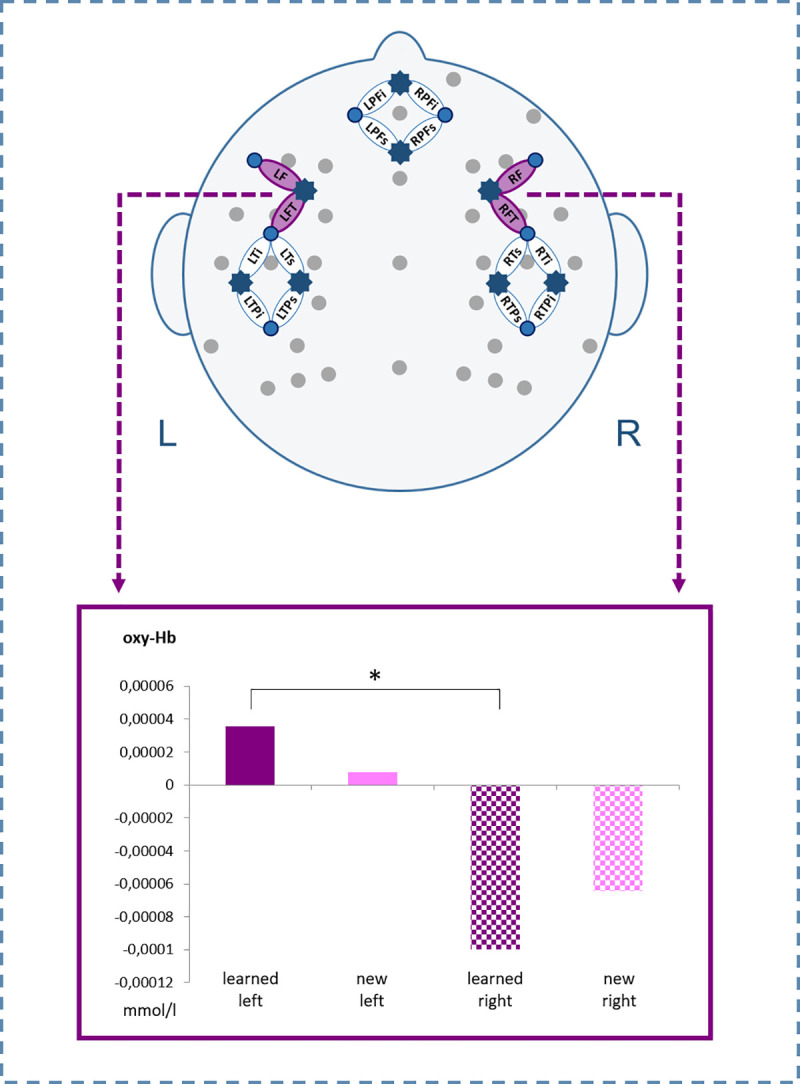
fNIRS results. Beta-values of [oxy-Hb] for new compared to learned pseudoword-picture pairings on left and right frontal ROIs (ø LF/LFT vs. ø RF/RFT). The asterisk indicates statistically significant differences.

The three-way ANOVA (Condition x Hemisphere x Region) did not reveal any significant main effect or interaction, neither for [oxy-Hb] nor for [deoxy-Hb]. The two-way ANOVA (Condition x Hemisphere) for [oxy-Hb] revealed a significant interaction for Condition x Hemisphere on the frontal region [*F*(1,30) = 4.413, *p* = .044, η_p_^2^ = .128]. Post-hoc *t*-tests showed stronger activations for learned PPPs only, on left (LF + LFT) compared to right frontal (RF + RFT) ROIs [*t*(30) = 2.783, *p* = .009; FDR corrected at *p*≤.025]. No significant effects were found for [deoxy-Hb] (see Fig 5).

#### 3.3.2 fNIRS results for correctly identified pseudoword-picture pairings

Neither the three-way ANOVA (Condition x Hemisphere x Region) not the two-way ANOVA (Condition x Hemisphere) resulted in any significant main or interaction effect for both [oxy-Hb] and [deoxy-Hb].

### 3.4 Follow-up behavioral production test

Results revealed that 34.9% of pictures were assigned correctly with the corresponding learned pseudoword. Interestingly, vowels (43.1%) and middle consonants (36.9%) were more often recalled correctly than onset consonant clusters (25.8%).

The evaluation of the adopted mnemonic strategies revealed that 58.3% of subjects predominantly remembered pairings based on similarities between pseudowords and words familiar from the native language (L1) (e.g., “fpata” sounds similar to “Panther” [German word for panther, engl.]), 20.8% of subjects predominantly focused on pictorial cues relevant from personal experiences (e.g., preference for certain food), and 12.5% of subjects could not indicate a specific strategy but reported the attempt to remember frequently appearing PPPs. 8.3% of subjects adopted both the first and second mentioned strategy.

## 4. Discussion

The present study aimed at investigating foreign language learning in adulthood by means of an implicit associative semantic training. This consisted in the repetitive presentation of congruous pseudoword-picture pairings (PPPs) intermixed with incongruous pairings during an implicit training without feedback. Importantly, pseudowords were specifically constructed to reflect a foreign language. Onset consonant clusters resembled phonotactic cues of the Slovak language, thus foreign language rules unknown/unfamiliar to the German-speaking subjects of the present study. The verification of learning success was tested by means of a recognition experiment in which subjects were confronted with congruous/learned PPPs from training alongside with totally new pairings. Here, they had the task to identify learned and new pairings. Such an implicit language learning design is new in the neuroscientific field. Fast dynamic processing mechanisms as well as recruited brain regions were assessed simultaneously by an innovative multi-methodological approach combining electroencephalography (EEG) as well as functional near-infrared spectroscopy (fNIRS).

From EEG data event-related brain potentials (ERPs) were analyzed. In this regard, results from the recognition experiment showed a larger negativity for new compared to learned PPPs. This effect was mainly distributed on midline electrodes and fronto-temporo-parietal electrode sites of the right hemisphere. This topographical distribution thus does not reflect a classical lexico-semantic N400 component [44] but mirrors an old/new effect [82–84]. The old/new effect is typically found during familiarity, memory and retrieval-related paradigms with the negativity usually being larger when subjects are confronted with new compared to old/known items (in literature often referred to as an increased positivity for old/known items). Furthermore, the old/new effect does not reflect a language-specific but a domain-general component (i.e., auditory, visual) being elicited also with stimuli from different semantic contexts (e.g., words, real objects, faces, etc. [83, 85–90]). However, research indicates that nonverbal stimuli such as meaningless objects often fail to elicit an old/new effect [86, 88, 91, 92]. The old/new effect was further found to differentiate between absolute and relative familiarity (for a recent review please refer to [50]). Absolute familiarity refers to pre-experimentally already established life-long knowledge, which applies to our presented pictures of known real objects. Relative familiarity, in contrast, indicates a recently acquired familiarity as it is the case for our completely unknown pseudowords conforming to foreign linguistic rules. However, relative familiarity also applies for pseudoword-picture pairings in a whole as they were recently presented during the semantic training directly prior to the recognition experiment. ERP studies addressing this dichotomy of familiarity found a more frontally distributed FN400 to index relative familiarity and a more posteriorly distributed N400 component to reflect absolute familiarity. A broad topographical distribution ranging from frontal to parietal areas as suggested by our ERP results therefore indicates that both aspects might have come into play.

Topographically, the old/new effect has been found to be differently distributed due to variations of task, study design, and material criteria. In particular, a right frontal old/new effect has been described as a mechanism of post-retrieval monitoring and decision making during a retrieval attempt (i.e., cognitive evaluation capacities necessitated by internal judgements prior to response selection) [82, 84, 93]. Such a mechanism might be the driving force also in our results, as subjects had to recognize earlier learned information explicitly, forcing them to actively make familiarity judgements. As ERPs did not show a purely linguistically driven process of an N400, results cannot confirm that phonotactic rules of a foreign language could successfully be integrated into the mental lexicon by changing from a non-word to a lexico-semantic status [47]. Instead, the results seem to indicate a more domain-general memory retrieval mechanism guided by familiarity. Intriguingly, this shows that adult subjects of the present study are indeed able to successfully memorize and recognize completely new foreign words assigned to a known picture even after such a short (i.e., 18 min) semantic training.

Furthermore, findings from our second neuroscientific method, the fNIRS, provide additional crucial insights about the nature of the underlying mechanisms. Results revealed that learned PPPs elicited larger activations on left frontal compared to homologous right hemispheric areas. This fact might point to the involvement of language-related processes, as the topography of these effects covers regions such as the ventro-lateral prefrontal cortex (vlPFC) including the inferior frontal gyrus (IFG).

Activations of the left IFG in particular were found during selection processes of semantic information when especially semantic competitors are present [32–35]. Even though subjects of our study have indeed to distinguish between learned and new PPPs, forcing them to actively select between stimuli, no explicit semantic or phonological competitors were present in the material. Thus, such an interpretation seems to be rather less likely for our effects.

The left IFG has also been found to be activated during processes of associative memory retrieval, especially in intentional learning settings compared to incidental ones (e.g., [94]). As our design represents both an intentional learning setting and an associative learning context in which participants had to build up associations between specific pseudowords and object pictures, such an interpretation seems to fit our results at least partially.

The IFG activation in our study was found during the recognition experiment in which subjects had to actively retrieve learned and new PPPs and thus had to control their selection/decision. We suggest that this activation might reflect top-down control processes postulated to be relevant especially during relative familiarity judgements, as such mechanisms are important for differentiating task-relevant from irrelevant information [50, 95, 96]. Thus, it seems highly plausible that they become active during our recognition experiment in particular for the selection of learned PPPs. The left dominance of this effect might be related to the mnemonic strategies used to better remember PPPs. A follow-up behavioral experiment in our lab with participants independently recruited from the neuroscientific experiments showed that 34.9% of pictures could be named correctly after the semantic training and recognition experiment. This means that subjects were able to successfully assign pseudowords to over one 3^rd^ of the previously learned pairings even after such a short semantic training. Interestingly, interrogation of subjects about their adopted memorizing strategies during semantic training further revealed a predominant use of mnemonic cues drawn from L1 (i.e., comparison of pseudowords with known words from L1) aiding the association between object and congruous pseudowords which supports mechanisms outlined by the Revised Hierarchical Model (RHM) [1]. This reliance on mnemonic cues from L1 might have contributed to the left dominance of the frontal activation found in the fNIRS.

A larger recruitment of left IFG has been shown in the context of language control, especially at the beginning of language learning (i.e., in low proficient learners) by the Convergence Hypothesis (CH) [39, 40]. During initial language learning, semantic retrieval in L2 is assumed to be more effortful and difficult than retrieval from L1, thus it necessitates increased controlled processes neurally reflected in enhanced frontal recruitment. Similar mechanisms could be plausible also for our study, as participants are at the very beginning of language learning and could reasonably have difficulties with controlling the judgement of learned compared to new PPPs. This might result in increased left IFG activation for learned PPPs as these are successfully recognized. A similar reliance on frontal areas in low proficient learners was also confirmed by functional MRI studies [41–43]. Interestingly, once semantic representations are better instantiated in long-term storage and participants thus become more proficient, this activation shifts to a more temporal recruitment, especially to the MTG. However, fNIRS findings of the present study did not show any temporal activation. This leads to the assumption that a short semantic training of 18 minutes does not yet enable learned words to be integrated in the mental lexicon. Most likely, rather initial domain-general control processes relevant for familiarity judgements are still at work at this early stage of vocabulary learning.

In addition, our robust findings (evidenced by large effect sizes) emphasize the importance of combining various neuroscientific methods as well as behavioral data. Using a multi-methodological approach enables each method per se to shed light on the neurophysiology and behavioral performance of learning. However, more importantly, both EEG and fNIRS bear the potential to complement each other in gathering substantial and profound information on mechanisms underlying language learning by assessing differential neurophysiological signals (i.e., electrophysiological and vascular responses). Furthermore, a simultaneous measurement provides the opportunity to investigate advantages as well as limitations of each single method. Interestingly, EEG and fNIRS revealed a differential sensitivity with respect to signal-to-noise. While EEG showed reliable results only for correctly identified PPPs, fNIRS resulted to be more stable when all (correctly and incorrectly identified) PPPs were analyzed. Recent studies showed that for extracting reliable ERPs, not only the mere number of trials [97] is important. ERPs are also impacted by the number of subjects and effect magnitude (defined in the magnitude of an effect in microvolts) [98]. Compared with their conclusions, our ERP effects are rather small in magnitude, however, we had a quite large number of subjects with an intermediate number of trials per condition. These factors are intertwined and possibly have led to the effect that ERPs only yield reliable effects when the signal is clean even though the number of trials is thus reduced. With respect to fNIRS, findings seem to indicate that here the number of trials per condition is crucial for ascertaining reliable results. However, further studies systematically varying different factors such as number of trials, number of subjects, and effect magnitude have to be conducted in order to provide essential insights in this regard. Another more theoretically driven explanation, at least for ERP data, is that familiarity effects usually are present only when items are identified correctly during the recognition task [50]. This further provides evidence that our ERP results resemble familiarity effects as they only occurred for correct responses and not when all responses were merged.

Our multi-methodological results nicely fit to assumptions recently proposed by the neurocognitive account of familiarity effects [50]. ERP findings predominantly suggest the involvement of relative familiarity with a contribution of absolute familiarity arising from known objects. fNIRS findings ascertain the influence of top-down control processes during the recognition task which is corroborated by results from the follow-up behavioral production task predominantly pointing towards the use of mnemonic cues from L1. Overall, results of the present study underline the fascinating plasticity of the adult brain during very initial foreign language learning, even after a short semantic training. The present study provides evidence that implicit associative learning paradigms are feasible and can be applied successfully in the neuroscientific field. Furthermore, we were able to show that phonotactics can be used to create solid foreign language learning materials. Finally, this study provides new evidence that relative and absolute familiarity play a crucial role during recognition tasks and are strongly intertwined with mnemonic and cognitive control mechanisms.
